# Age, Risk, and Life Expectancy in Norwegian Intensive Care: A Registry-Based Population Modelling Study

**DOI:** 10.1371/journal.pone.0125907

**Published:** 2015-05-26

**Authors:** Frode Lindemark, Øystein A. Haaland, Reidar Kvåle, Hans Flaatten, Kjell A. Johansson

**Affiliations:** 1 Department of Research and Development, Haukeland University Hospital, Bergen, Norway; 2 Department of Global Public Health and Primary Care, University of Bergen, Bergen, Norway; 3 Norwegian Intensive Care Registry, Helse Bergen HF, Bergen, Norway; 4 Department of Anesthesia and Intensive Care, Haukeland University Hospital, Bergen, Norway; 5 Department of Clinical Medicine, University of Bergen, Bergen, Norway; Karolinska Institutet, ITALY

## Abstract

**Background:**

Knowledge about the expected life years gained from intensive care unit (ICU) admission could inform priority-setting decisions across groups of ICU patients and across medical specialties. The aim of this study was to estimate expected remaining lifetime for patients admitted to ICUs during 2008–2010 and to estimate the gain in life years from ICU admission.

**Methods:**

This is a descriptive, population modelling study of 30,712 adult mixed ICU admissions from the Norwegian Intensive Care Registry. The expected remaining lifetime for each patient was estimated using a decision-analytical model. Transition probabilities were based on registered Simplified Acute Physiology Score (SAPS) II, and standard and adjusted Norwegian life-tables.

**Results:**

The hospital mortality was 19.4% (n = 5,958 deaths). 24% of the patients were estimated to die within the first year after ICU admission in our model. Under an intermediate (base case), optimistic (O), and pessimistic (P) scenario with respect to long-term mortality, the average expected remaining lifetime was 19.4, 19.9, and 12.7 years. The majority of patients had a life expectancy of more than five years (84.8% in the base case, 89.4% in scenario O, and 55.6% in scenario P), and few had a life expectancy of less than one year (0.7%, 0.1%, and 12.7%). The incremental gain from ICU admission compared to counterfactual general ward care was estimated to be 0.04 (scenario P, age 85+) to 1.14 (scenario O, age < 45) extra life years per patient.

**Conclusions:**

Our research demonstrated a novel way of using routinely collected registry data to estimate and evaluate the expected lifetime outcomes for ICU patients upon admission. The majority had high life expectancies. The youngest age groups seemed to benefit the most from ICU admission. The study raises the question whether availability and rationing of ICU services are too strict in Norway.

## Introduction

Demand for intensive care usually exceeds the availability of such treatment [1–3]. Aging populations and shifting disease patterns have led to an increase in the demand for intensive care services in many countries. Treatment in intensive care units (ICU) is generally considered to be expensive and capacity needs to be increased substantially over coming decades if health systems are to meet increasing demands. Additionally, intensive care services may need to be prioritised between patients. Ideally, such priorities ought to be supported by explicit and acceptable criteria such as cost-effectiveness, severity of disease, risk estimates, age, or expected lifetime health gains [4–6].

Priority setting can be defined as providing potentially beneficial treatment to some individuals or patient groups. When resources are limited, this involves withholding treatment from other patients [7–10]. In order to obtain a fair and efficient allocation of health care resources, two major principles for priority setting must be considered. According to a health maximising principle, intensive care should be allocated towards interventions that attain the greater expected health in the population [11–13]. Another principle, motivated by a concern for fairness, is that patients in equal situations should be given equal opportunities [14]. To set and evaluate ICU priorities rationally and coherently, we need evidence based information about the size of expected health benefits from intensive care [15].

A number of studies report the short- or long-term mortality in patients admitted to intensive care. Several studies indicate poor outcomes for old patients admitted to ICUs [16]. This has raised questions about the benefit of ICU admission for old patients [17]. One suggestion has been to offer treatment in intermediate units or general wards rather than ICUs for patients who may benefit similarly from care in less resource-demanding settings. Another concern has been the value of high-cost life-extending interventions at the end of life. For example, in the UK the National Institute for Health and Care Excellence is currently using an end of life criterion to modify the cost-per-quality-adjusted life year [18].

Most studies evaluating the outcome of intensive care only include patients admitted to the ICU. As a consequence, we know less about health outcomes of potential ICU patients not being admitted to the ICU. The alternatives to ICU admission and the associated health outcomes are often poorly specified for the population at risk [5,19,20]. Randomisation of patients to ICU admission vs. general wards in a clinical trial is ethically controversial [5]. Interestingly, a large European multi-centre study published in 2012 (ELDICUS II) was the first study to demonstrate a mortality benefit in those accepted vs. refused admission, where the short-term benefit appeared to be much higher in older patients than younger patients [21]. The authors concluded that physicians should reconsider admission practices for the elderly, since studies have demonstrated that intensive care is more frequently withheld from older than younger patients [16,17,22]. However, the study did not take into account the expected remaining lifetime of the patients.

The feasibility of using mortality prediction models and/or life expectancy to prioritise patients in intensive care has been the subject of some discussion, but knowledge about this is scarce and needs further analysis [1,3,21,23,24]. At hand information about the expected remaining lifetime of ICU patients and the benefit of ICU admission in terms of life years gained could inform decisions about efficient and fair prioritisation of resources, both across groups of ICU patients and across medical specialties.

Almost all ICU patients in Norway are registered in the Norwegian Intensive Care Registry (NIR). In this paper we seek to describe the expected lifetime outcomes for ICU patients > 18 years old based on risk estimates and age with data available from NIR. We hypothesise that a substantial proportion of ICU patients have poor prognosis and benefit marginally from ICU admission. Our aim is to develop a systematic method able to estimate expected remaining life years at the point of admission to the ICU, and to estimate the gain from ICU admission compared to the hypothetical rejection of ICU admission and treatment in a general ward.

## Methods

### Study design, setting and study population

We performed a descriptive combined registry-based modelling study, where primary data were drawn from NIR. NIR is a national medical quality registry containing routinely collected data, and covers more than 90% of adult Norwegian ICU admissions. In 2008–2010 the registry received individual patient records from 42 surgical, medical or mixed ICUs in 38 hospitals at all levels of our health care system [25–27]. Patients transferred from one ICU to another or re-admitted to the ICU during the same hospital stay were excluded to avoid counting the same individual more than once. The source population consisted of all patients registered in NIR during 2008–2010. NIR represents a general ICU population, and is characterized by case-mix (heterogeneity in terms of, e.g., diagnosis, co-morbidities, or specific treatment needs).

### Ethics statement

The NIR Steering group and the data protection officer at Haukeland University Hospital approved our access to and analysis of the anonymous data set. REC West, the Regional Committee for Medical and Health Research Ethics has waived approval because of the non-interventional nature of the study and the use of anonymous data only.

### Variables

For each individual patient we abstracted data on age, gender, simplified acute physiology score II (SAPS II), type of admission, hospital category, length of ICU stay (LOS), and vital status on discharge from ICU and from hospital. SAPS II score has been validated in several ICU populations including Norwegian ones [28–32]. SAPS II generally overestimates the hospital mortality [29,30].

### Model

We developed a model that predicts the remaining life expectancy for each NIR patient at the point of ICU admission.

#### Probability of survival to hospital discharge

**Fig 1:**
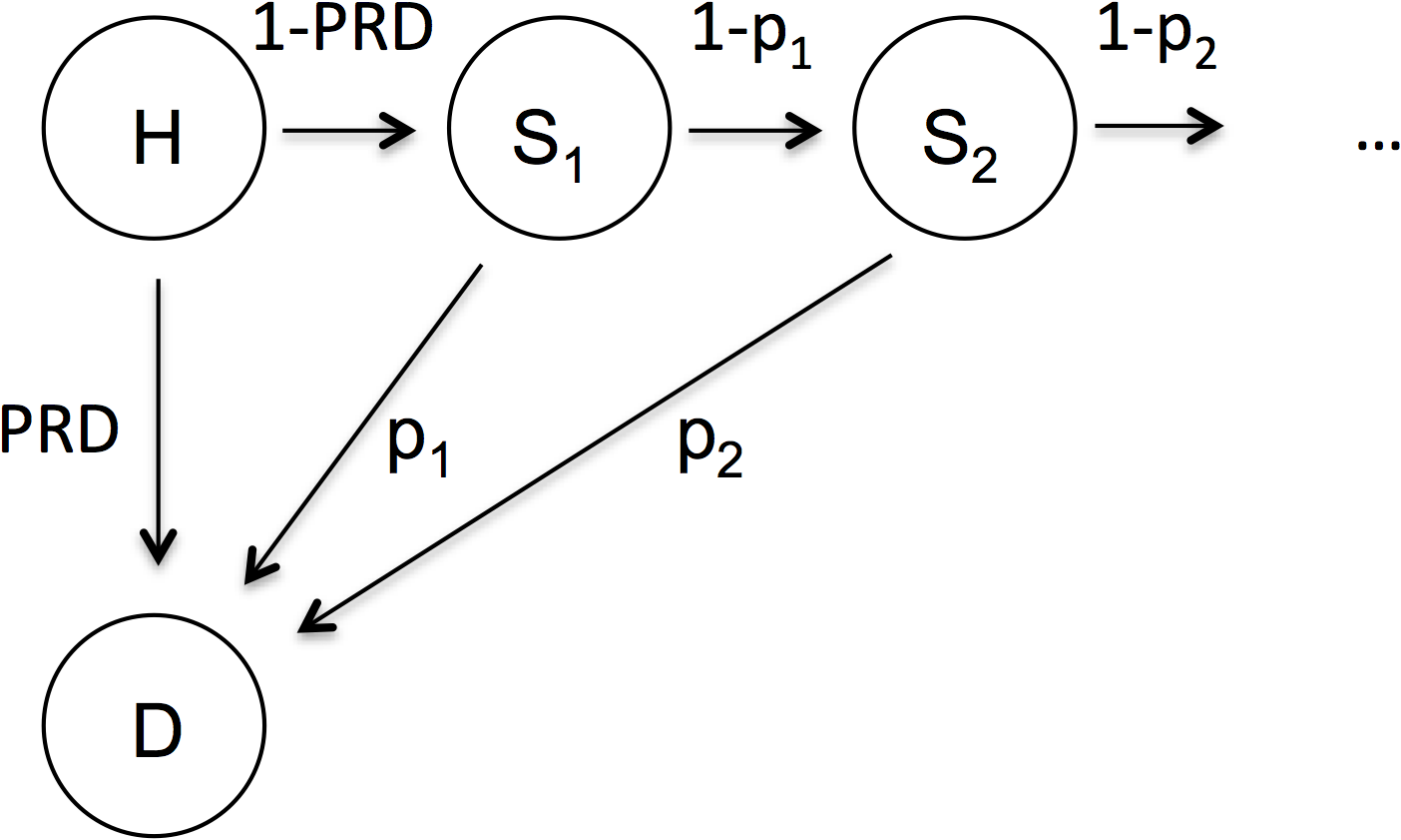
State transition diagram. H (alive hospital), D (dead), and S_x_ (alive at age x, post hospital discharge) represent the health states. PRD is the predicted risk of death during hospitalisation, and p_x_ is the probability of death at age x derived from the Norwegian life table 2011.

In step one, the predicted short-term risk of death (PRD) during hospitalization was calculated based on SAPS II for each patient. Note that the probability for surviving the hospital stay is 1-PRD (Fig 1). In order to improve the estimates of PRD, a standard logistic regression analysis was used to calibrate the original SAPS II with the observed hospital mortality in the NIR data set. Details on this calibration procedure has been published elsewhere [33].

#### Life expectancy: base case

In step two, we calculated the age-specific life expectancy (aLE) using standard life-table methodology to project survival into the future for those surviving the hospital stay [34]. Conceptually, this corresponds to constructing a two-state Markov model, where patients can be in in a health state “alive” or in a health state “dead” [35]. aLE was based on a Norwegian life table 2011, using age-specific mortality data in the general population for both sexes (Fig 1 and S2 File) [36]. Hence, for an individual, i, admitted to the ICU the expected remaining life years (ELYi) could be calculated using the formula

$${ELY}_{i}=(1-{PRD}_{i})\times {aLE}_{i}$$ (1)

The outcome of the model was the undiscounted expected remaining life years (ELY_i_) for each patient in the study population.

Several studies show that ICU survivors have excess mortality compared with the general population in the years following hospital discharge [37]. aLE was adjusted to take this excess long-term mortality into account by including model parameters K and L as follows: First, the standard age-specific annual mortality rate was increased K-fold the first year after hospital discharge. Second, after decreasing for L years, the further mortality of the ICU survivors was assumed to be back at the level of the general population. Third, the rate of decrease was assumed to be linear. The choice of a linear decrease in the excess mortality was arbitrary. In the base case we chose K = 3 and L = 3, reflecting excess mortality found in previous studies [37–41]. These base case assumptions result in a survival curve in our model that resembles the findings in a recently published review of observational studies of the long-term mortality in general ICU populations [37]. National data on long-term mortality in former ICU patients are lacking in Norway.

**Fig 2:**
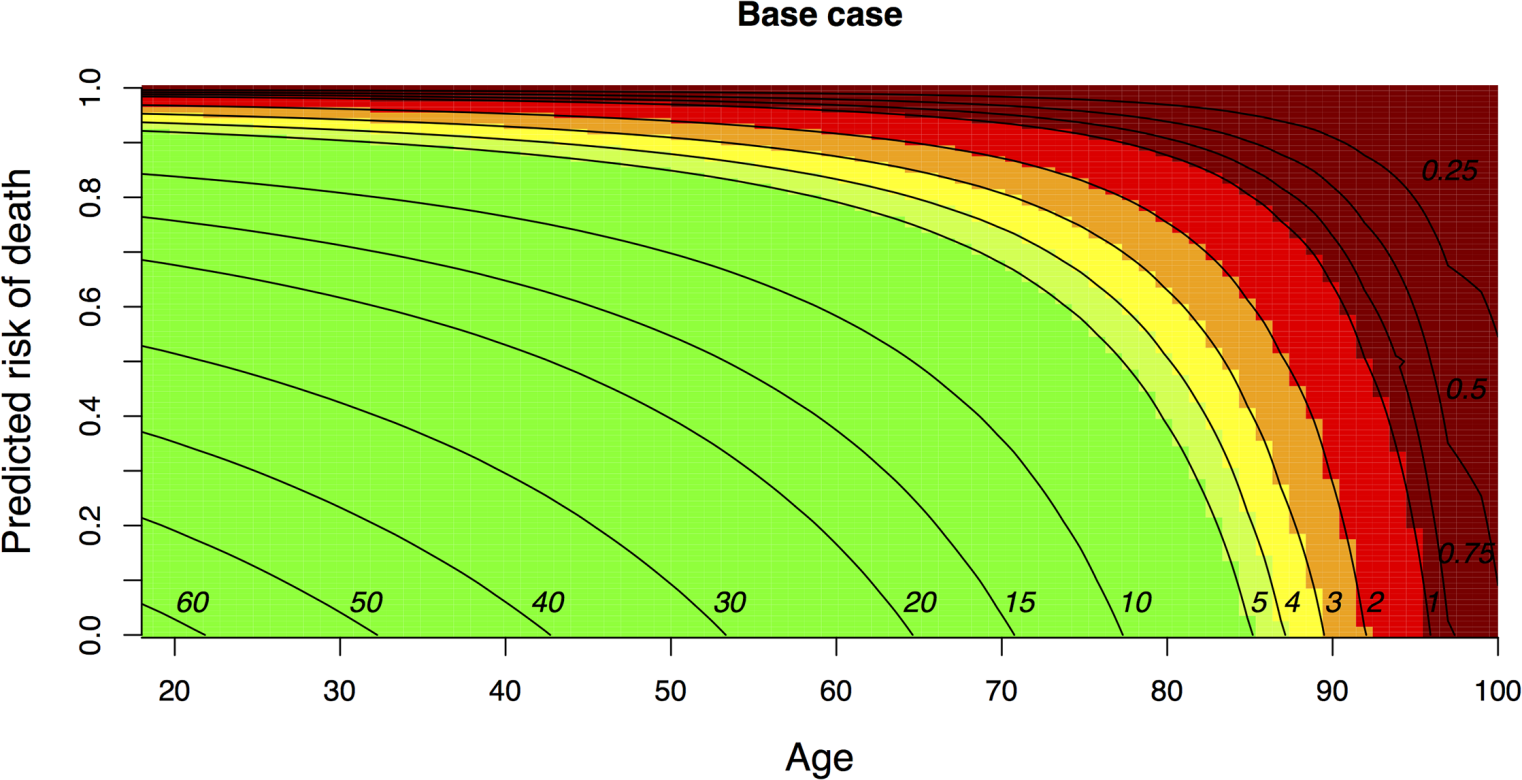
Outcome chart. The expected outcome by the short term predicted risk of death and age. The remaining life expectancy is indicated at the right end of the contours. Colours signify long to short life expectancy: green, > 5 years; light green 4–5 years; yellow, 3–4 years; orange, 2–3 years; red, 1–2 years; dark red, < 1 year).

The outcome chart illustrates the framework we used to estimate the expected outcome for a patient in the data set (Fig 2). The outcome chart is generic where age and the predicted short-term risk of death determine the position and coordinates of each patient in the dataset.

We categorized the patients according to the estimated remaining lifetime (ELY_i_) and show the magnitude and distribution of the expected health outcomes for all patients and by type of admission (medical, acute surgical, or planned surgical).

#### Life years gained from ICU admission

In step three, we estimated the mortality reduction of accepting vs. rejecting ICU admission in a counterfactual scenario. We sought to illustrate the expected incremental gain in life years from ICU admission compared with treatment in a general ward. To that end, we needed to compare the PRD of a patient from the NIR population, with PRD_rejected_, the predicted short-term risk of death of a hypothetical patient with the same SAPS II being denied intensive care. Like most ICU registries, NIR does not contain data on patients who are referred and rejected to the ICU. Sprung *et al*. have provided observational data on short-term mortality for specific age-groups of patients who were rejected and patients who were accepted to the ICU, based on a large prospective European multi-centre study of ICU triage decisions [21]. Based on these data we calculated the relative risk, RR, which expresses the short-term mortality benefit of ICU admission. RR ranged from 0.71 to 0.95: 0.82 (age 18–44), 0.95 (age 45–64), 0.81 (age 65–74), 0.88 (age 75–84), and 0.71 (age 85+). The magnitude of the short-term mortality benefit of ICU admission is uncertain. In the base case, we chose an intermediate RR = 0.8. In a one-way sensitivity analysis we repeated the calculations with RR = 0.7 and RR = 0.9.

Knowing RR, it follows that for a patient from the NIR population, we have PRD_rejected_ = PRD_i_/RR. The upper boundary for PRD_rejected_ was set to one, because the probability of death cannot exceed 100%. By substituting PRD with PRD_rejected_ (Fig 1), we could estimate the expected remaining life years for each patient if hypothetically rejected to the ICU. The gain from ICU admission is the difference in the expected life years when being admitted vs. rejected to the ICU. We report the estimated incremental gain in life years from ICU admission across five age groups.

### Life expectancy: scenario analysis with respect to long-term mortality

Recognizing that ICU patients are a diverse group with varying long-term survival and due to lack of long-term survival estimates in NIR patients, we added two scenarios that differ from the base case with respect to the risk of death after hospital discharge. An optimistic scenario, Scenario O, assumes an age specific long-term mortality among ICU survivors that is equal to the general population, so that K = 1 and L = 1.

In a pessimistic scenario, Scenario P, we set K = 8 and L = 30, which means that the age-specific annual mortality rate was increased eight-fold the first year after hospital discharge, and did not reach the level of the general population until 30 years after discharge.

#### Survival curve

In order to elucidate the relationship between life expectancy and mortality rates, we plotted the projected survival curve for the 10 years following admission for all three scenarios, and for the base case by type of admission.

The statistical analysis and life table calculations were performed using R statistical software version 3.0.2 [42]. 95% confidence intervals for expected remaining life years and life years gained from ICU admission were obtained using a bootstrap approach with 1,000 replications. In each replication, 30,712 individuals were sampled with replacement from the original data set. The 95% confidence limits were selected using the 2.5 percentile and 97.5 percentile from the 1,000 replications.

## Results

### Study population

**Fig 3:**
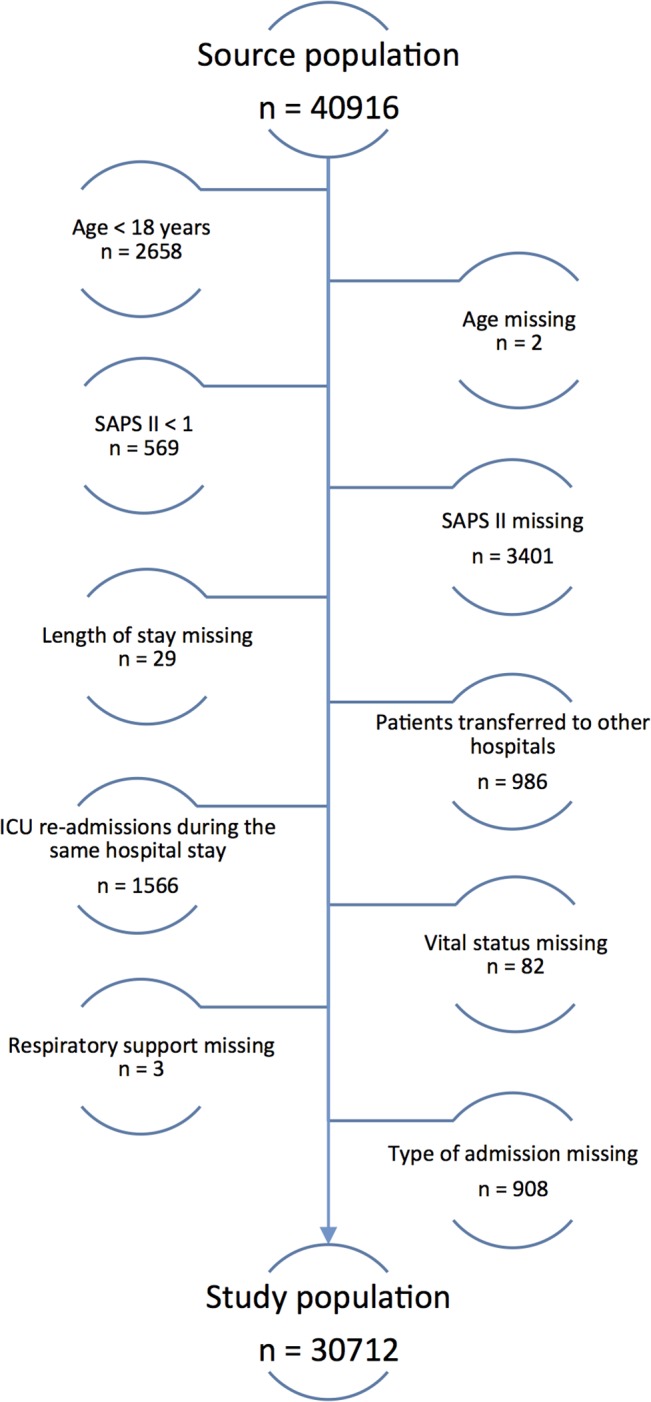
Patient selection. Order of exclusion of patients for the purpose of our study. The source population constitutes all patient records in the National Intensive Care Registry in the years 2008, 2009 and 2010. The numbers refer to patients who were excluded from our analysis.

The total number of registered ICU admissions was 40,916. Patient exclusion criteria are outlined in Fig 3, where the final study population was 30,712. Key characteristics of the patients are shown in Table 1. There were insignificant differences between the source and the study populations. 19.4% of the patients in the study population died during the hospital stay. The hospital non-survivors were on average older, had higher SAPS II scores, and were more often admitted for medical reasons than the survivors. Of these non-survivors, 66% died in the ICU, 34% died in wards after discharge from the ICU.

**Table 1:** Patient characteristics.

Patient characteristic	All patients > 18 years	Sample	Hospital survivors	Hospital non-survivors
n total	38258	30712	24754	5958
Age (years)				
Missing	2	-	-	-
Mean (sd)	62.7 (18.2)	63.2 (18.2)	61.3 (18.4)	71 (14.9)
Q1	51.9	52.4	49.7	63
Median	65.6	66	64.1	74.1
Q3	76.9	77.3	75.7	82
Sex				
Missing	3	-	-	-
Female	0.43	0.43	0.43	0.43
SAPS				
Missing	3403	-	-	-
Mean (sd)	36.2 (18.5)	36.8 (18.2)	32.5 (15.3)	55 (18.3)
Q1	23	24	21	41
Median	34	34	31	54
Q3	47	47	42	67
LOS (days)				
Missing	3	-	-	-
Mean (sd)	4.2 (6.8)	4.3 (6.8)	4.1 (6.5)	5 (8.1)
Q1	1.1	1.1	1.1	0.8
Median	1.9	2	2	2
Q3	4.2	4.3	4.1	5.4
Type of admission				
Missing	1962	-	-	-
Medical	0.53	0.56	0.54	0.64
Acute surgery	0.3	0.32	0.32	0.3
Planned surgery	0.18	0.13	0.14	0.06
Hospital category				
Missing	2	-	-	-
Primary	0.34	0.37	0.38	0.32
Secondary	0.38	0.4	0.39	0.44
Tertiary	0.28	0.24	0.23	0.24
Survival status				
Missing	1801	-	-	-
Died ICU	0.12	0.13	0	0.66
Died ward	0.06	0.07	0	0.34
Survived hospital	0.82	0.81	1	0

Characteristics of all the patients aged 18 years or over admitted to an ICU and registered in the Norwegian Intensive Care Registry in 2008, 2009 and 2010, patients selected for this study and the hospital survivors and non-survivors in the study population.

### Life expectancies

**Fig 4:**
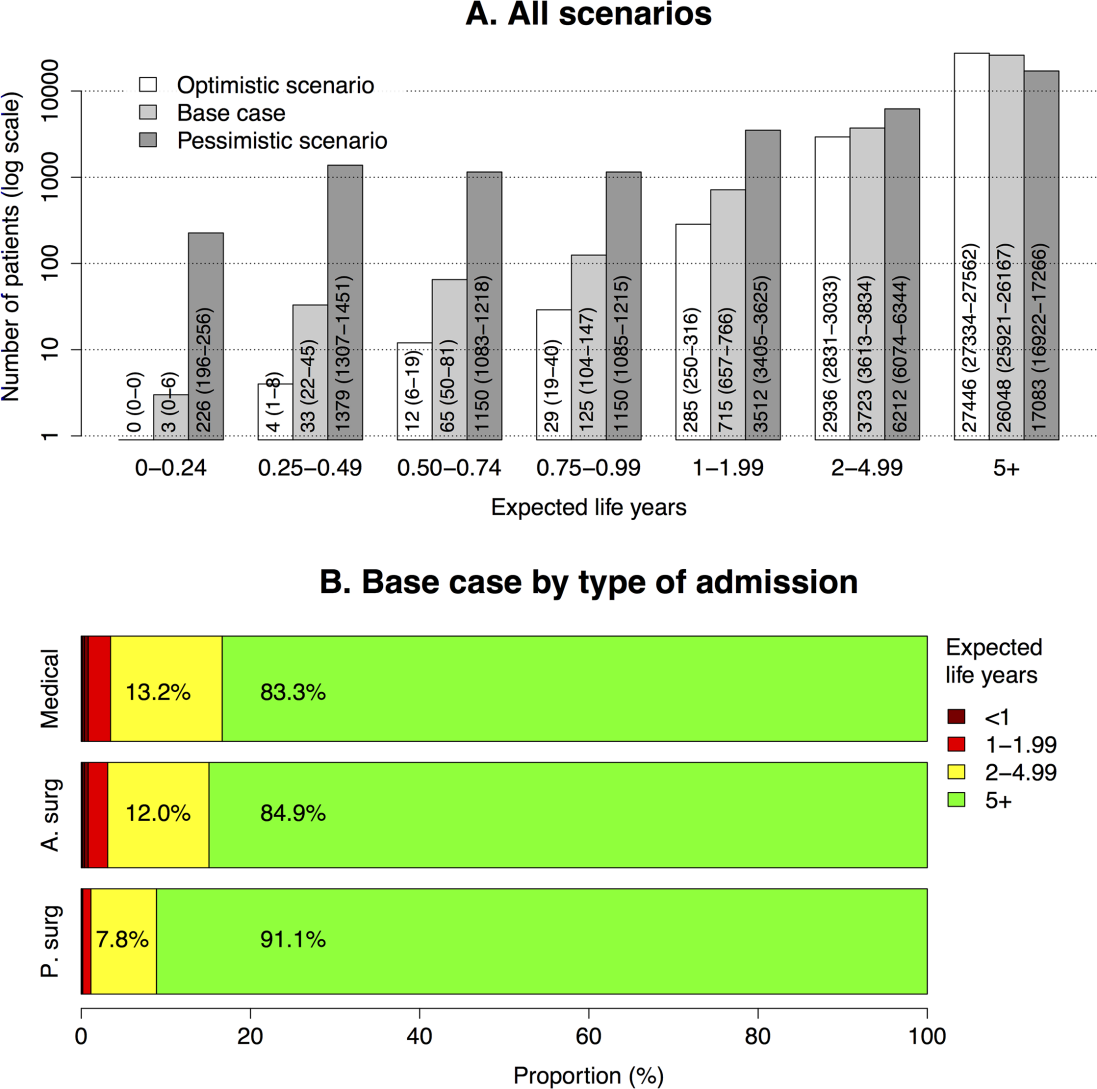
Expected remaining lifetime. A. Number of patients (95% confidence intervals) in categories defined by the size of the expected outcome in terms of expected remaining life years. Three different scenarios in each category regarding the calculation of post hospital mortality: according to a life table with moderately high long-term mortality (base case), a standard life table (Scenario O), and life table with very high long-term mortality (Scenario P). B. Proportion of patients by type of admission: planned surgical, acute surgical, and medical (base case).

The average expected remaining lifetime upon admission was 19.4 years (base case). Fig 4A shows the distribution of the size of the expected outcome among all the patients. The number of patients with a life expectancy of at least five years was 26,048 (84.8%). Few patients had an expected remaining lifetime of less than one year (n = 226, 0.7%) and of less than six months (n = 36, 0.1%). The proportion of patients with an expected remaining lifetime of 2 years or more were 96.5% among medical, 96.9% among acute surgical, and 98.9% among planned surgical admissions (Fig 4B).

### Life years gained from ICU admission

The expected gain in life years from ICU admission vs. rejection is shown in Table 2. The youngest age group had the highest expected gain from admission, 1.14 life years on average. The expected gain from admission decreased with older age groups, to an estimated average of 0.22 life years gained among those aged 85+ (base case). These estimates ranged from 0.51–1.93 (age < 45) to 0.10–0.37 (age 85+) life years gained in the sensitivity analysis.

**Table 2:** Expected benefit: life years gained from ICU admission.

		Base case	
	RR = 0.7	RR = 0.8	RR = 0.9
18–44	1.93 (1.86–2.01)	1.14 (1.09–1.18)	0.51 (0.49–0.53)
45–64	1.87 (1.83–1.91)	1.11 (1.09–1.14)	0.50 (0.49–0.51)
65–74	1.34 (1.31–1.37)	0.80 (0.78–0.82)	0.36 (0.35–0.37)
75–84	0.84 (0.82–0.86)	0.50 (0.49–0.51)	0.23 (0.22–0.23)
85+	0.37 (0.36–0.38)	0.22 (0.22–0.23)	0.10 (0.10–0.10)

Average life years gained (95% confidence intervals) from ICU admission vs. hypothetical rejection and care in a general ward across different age groups. Relative risk (RR) based on observed mortality in accepted vs. rejected patients in the Eldicus II study.

### Scenario analysis with respect to long-term mortality

The average expected remaining lifetime was 19.9 years in scenario O. The expected remaining lifetime was less than six months in 4 cases (0.01%), and less than one year in 45 cases (0.1%). The number of patients with a life expectancy of at least five years was 27,446 (89.4%) (Fig 4A).

In Scenario P, the average expected remaining lifetime was 12.7 years. The expected remaining lifetime was less than six months for 1605 patients (5.2%), and less than one year for 3905 patients (12.7%). The number of patients with a life expectancy of at least five years was 17,083 (55.6%) (Fig 4A).

In Table 3, the estimates of the expected gain in life years are reported as the range between the pessimistic and optimistic scenarios.

**Table 3:** Life years gained from ICU admission in a range from Scenario P to Scenario O.

	Gain (LYs)
18–44	1.01 (0.97–1.05)- 1.14 (1.10–1.18)
45–64	0.65 (0.63–0.66)- 1.13 (1.10–1.15)
65–74	0.30 (0.30–0.31)- 0.84 (0.82–0.85)
75–84	0.13 (0.13–0.14)- 0.57 (0.56–0.58)
85+	0.04 (0.04–0.04)- 0.32 (0.31–0.33)

Average life years gained (95% confidence intervals) from ICU admission vs. hypothetical rejection and care in a general ward across different age groups. Relative risk (RR = 0.8) based on observed mortality in accepted vs. rejected patients in the Eldicus II study.

### Survival curve

**Fig 5:**
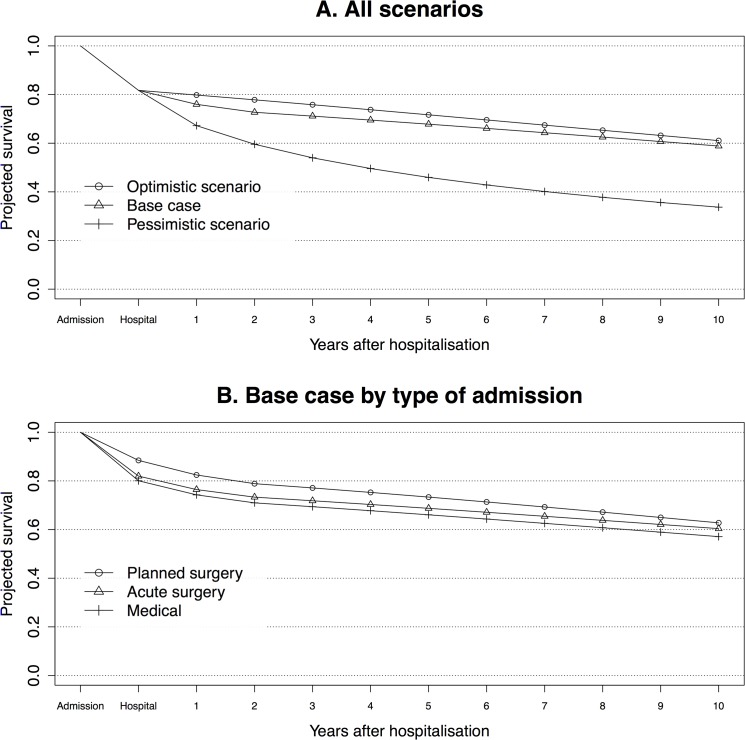
Projected survival. A. Survival of the modelled ICU population at ICU admission (admission), hospital discharge (hospital) and subsequent years. Three different scenarios of post hospital mortality are illustrated in each time cycle: according to a life table with moderately high long-term mortality (base case), a standard life table (Scenario O), and life table with very high long-term mortality (Scenario P). B. Survival of the modelled ICU population by type of admission (base case).

Fig 5A shows the estimated survival curve from our model, with the three different scenarios after hospital discharge. During the hospital stay, 5,616 (18.3%) were estimated to die and 25,096 to survive. Note that this is somewhat lower than the observed number of deaths (19.4%). This is because the estimate is based on the NIR SAPS II model, which was calibrated on a subset of our study population [43]. As indicated by the survival curve, 24% were estimated to die within the first year after ICU admission in our model (base case). For patients aged 80+, the 1-year mortality after admission was estimated to be 43% in Scenario O, and 71% in Scenario P. Fig 5B illustrates that the hospital mortality predicted by SAPS II was much lower among planned surgical admissions (11.6%) than among acute surgical (18.0%) and medical (19.9%) admissions. Fig 5 was created using exactly the same mortality rates as those used to calculate the life expectancy.

## Discussion

We developed a decision-analytical model that predicts the life expectancy of patients admitted to ICUs. Overall, the results of this registry-based modelling study indicate that intensive care resources are allocated towards patients with good expected outcomes regarding survival. Most adults that were admitted to Norwegian ICUs in 2008–2010 had high remaining life expectancy. About 1% of those admitted had a life expectancy of less than one year after admission to the ICU. These findings stand in contrast to the fact that 24% of patients in the model were expected to die within the first year after admission. These findings illustrate an important difference between longevity of life and mortality rates as outcome measures. The former draws an optimistic picture and the latter shows more pessimistic results, whereas both metrics derive results from the same dataset. ICU admission was estimated to extend life between 0.04 (Scenario P, age 85+) and 1.14 (Scenario O, age < 45) life years, compared to counterfactual care in a general ward.

To our knowledge, this is the first study to describe the life expectancies in a national general ICU population in any Nordic country. Few studies have estimated the expected remaining life years at admission by combining patient-level information, mortality prediction models, and life tables. One advantage with this approach is that the expected lifetime outcomes can be estimated even if there is scarce data on observed mortality. Potentially, a generic outcome model may apply short-term death risks from other mortality prediction models than SAPS II, such as APACHE II, or EuroSCORE for candidates for cardiac surgery. In specific patient populations and possibly in individual patient decision-making, where there is more information about the determinants of the short- and long-term prognosis, clinicians can use the outcome chart (Fig 2) guided by good clinical judgement to inform value discussions about expected lifetime outcomes for their patients. However, such generic modelling tools can never replace clinical discretion, but potentially provide useful collateral information. Some studies have reported the life expectancies in different subgroups of the ICU population, but we have not seen similar studies that rank the patients according to the size of the lifetime outcomes [44].

The majority of patients had a life expectancy of more than one year and ICU admission appeared to extend life between 0.2 and 1.1 life years compared to care in a general ward (base case). These findings contradict the perception that we are allocating scarce and expensive intensive care resources towards too many patients with poor prognosis and marginal potential benefits. For comparison, priorities should be consistent across specialities and the value of high-cost interventions that can prolong life for a few months at the end of life should be equal [18]. If a patient with a given prognosis without treatment and a certain expected benefit from treatment is assigned a high priority within one specialty, another patient in the same situation in a different specialty should be treated similarly [14]. For example, patients with advanced cancer with an estimated life expectancy of one year *including* a health gain of 3–6 extra months from high-cost, advanced chemotherapy, are usually offered such treatment in oncology departments [45]. According to a principle of equal treatment, in this situation the cancer patients should have the same priority as ICU patients with a short life expectancy, because they have similar prognosis without and with intervention [14]. Since our results suggest that relatively few ICU patients, about 1%, are in this category, our study raises the question whether the availability and priority setting of ICU services are too strict compared to other services. Especially as we know that the number of ICU beds in Norway is low [46].

Younger patients had lower predicted short-term risk of death, and smaller absolute risk difference between accepted vs. hypothetically rejected patients. Still, the gain in life years was higher among the youngest compared with the oldest age group. On a lifetime scale, the greater mortality reduction among older patients appears to be offset by the fact that they have already had many life years (Fig 2). From a strict health maximizing point of view, therefore, resources should be directed towards the younger [47]. The estimates of the life years gained from ICU admission were sensitive to the methods used to calculate the short-term mortality benefit from being accepted vs. refused ICU admission.

Few studies report expected lifetime outcomes for ICU patients. Previous literature usually report the long-term outcome for ICU patients as the mortality or survival rate at a certain time after ICU admission or hospital discharge [37]. The average age and life expectancy in our study resemble the estimates in the study by Edbrooke *et al*., who found that the average life expectancy was close to 15 years based on the patient cohort in Eldicus II [19]. They used country-specific life tables and adjusted for excess mortality the first four years post hospital discharge.

Strengths of our study include a large study population with subjects across relevant age groups, which we could use to build a generic outcome model based on patient-level data.

Selection bias due to missing data on SAPS II may affect the internal validity of the study. However, the distribution of key factors such as age was similar in the study population and in the excluded patients with missing SAPS II. It is therefore unlikely that the validity of the study has been strongly affected. Uncertainty about potential variation in scoring practices among ICU staff and between various ICUs may reduce the precision of our estimates [25–27].

The impact of individual factors that are known to be associated with higher mortality after discharge such as co-morbidities, reason for admission and diagnosis, and physical and cognitive function is not well captured in the analysis. NIR does not contain such data [37]. At the individual level, therefore, the life expectancy is almost certainly overestimated for some patients. When speaking of the ICU population as a whole, we compensate for such case-mix associated impacts on long-term survival by also reporting results in a range between an optimistic and pessimistic scenario. Of course, elective surgical patients, who constituted a smaller proportion in our study population compared with many other general ICU populations (13% of the study population, 6% of the hospital non-survivors, 3–8% of those with ELY_i_<1), usually have better short- and long-term survival than medical and acute surgical patients [48]. This is illustrated by Fig 4B showing a smaller proportion of patients with short life expectancy among planned surgical than acute surgical and medical admissions. Their long-term mortality may lie between the base case and Scenario O, whereas the long-term mortality in for example older ICU patients with circulatory failure may approximate that of the pessimistic scenario (Fig 5) [49].

Another important limitation is that we, like most other investigators, lack information about the outcomes in a comparator group of patients potentially eligible for admission but not admitted. That is why we adopted estimates of the benefit of admission from Sprung *et al*., and the limitations of that study of course apply to the estimates of the benefit of admission in our study [21]. The mortality benefits in our model are based on study patients in Eldicus II who were refused admission because the ICU was full, or the patients were judged to be too sick or too well to benefit. Therefore, the gain in life expectancy we report may express the benefit in a selected group for which the most difficult admission choices are being made, and be an underestimate of the benefit in a majority of the ICU population.

## Conclusions

This paper demonstrated a novel way of using routinely collected registry data to estimate and evaluate, in a lifetime perspective, the expected outcome for patients admitted to ICUs. Overall, intensive care resources in Norway seem to be allocated towards patients with good expected lifetime outcomes. Patients with short life expectancy appear to comprise a relatively small proportion of ICU admissions. The gain in life years from ICU admission seems to be comparable with gains from high-cost interventions offered in other fields. The study raises the question whether the availability and rationing of ICU services are too strict in Norway. We provided an outcome chart that may facilitate ethical discussions on the value of expected lifetime outcomes in the context of medical priority setting. The outcome model can be developed further to study the cost-effectiveness of intensive care.

## Supporting Information

S1 File Norwegian Intensive Care Registry dataset 2008–2010. (CSV)

S2 File Life table Norway 2011. (XLSX)
